# Supplementary material for: Planispine A Sensitized Cancer Cells to Cisplatin by Inhibiting the Fanconi Anemia Pathway
Source: Molecules. 2022 Oct 26;27(21):7288. doi: 10.3390/molecules27217288 (PMC9654912; doi:10.3390/molecules27217288)
Supplement: Supplementary file 1 [file molecules-27-07288-s001.zip › molecules-1932902-supplementary.pdf]

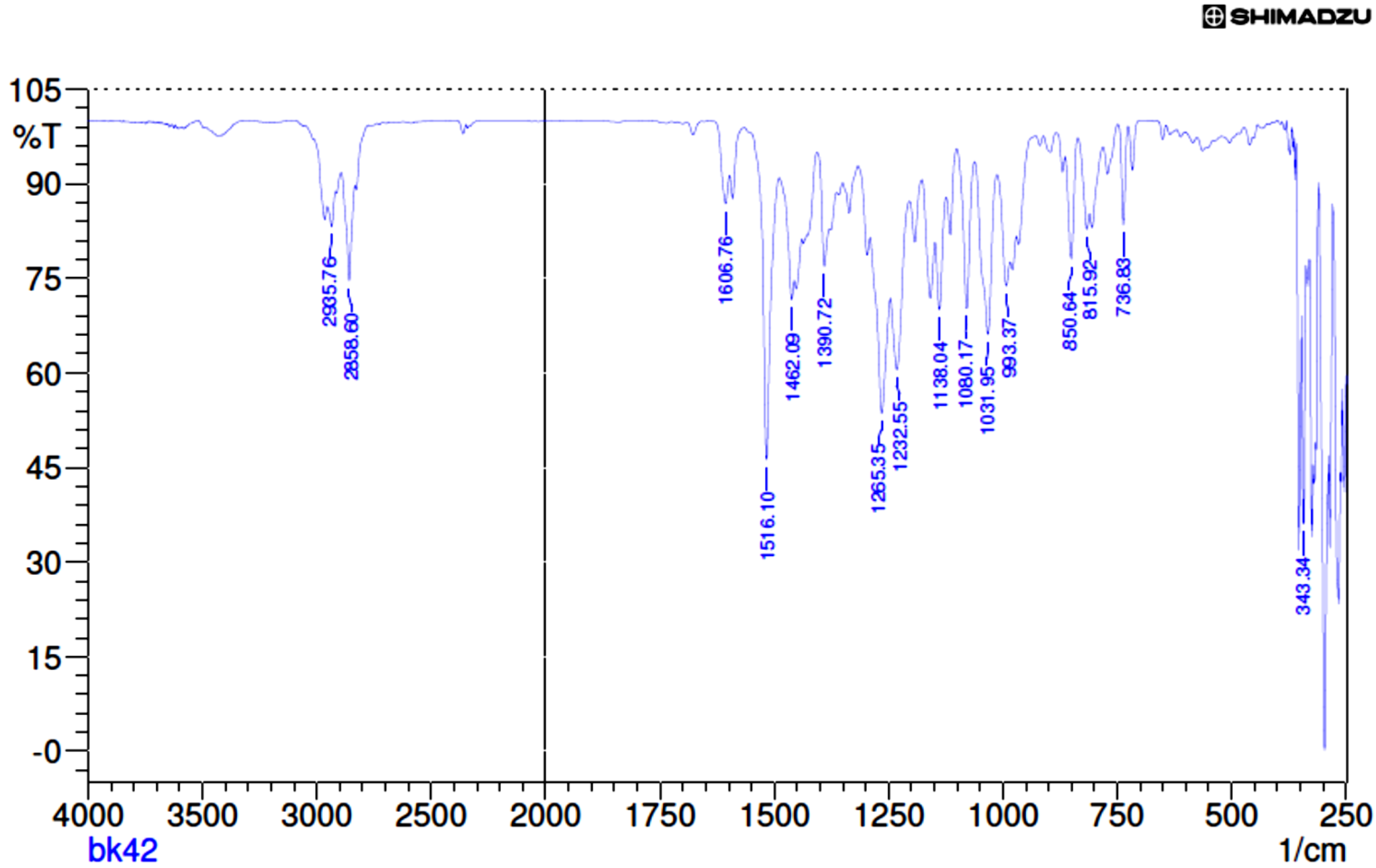

Figure S1: FTIR spectrum of planispine A.

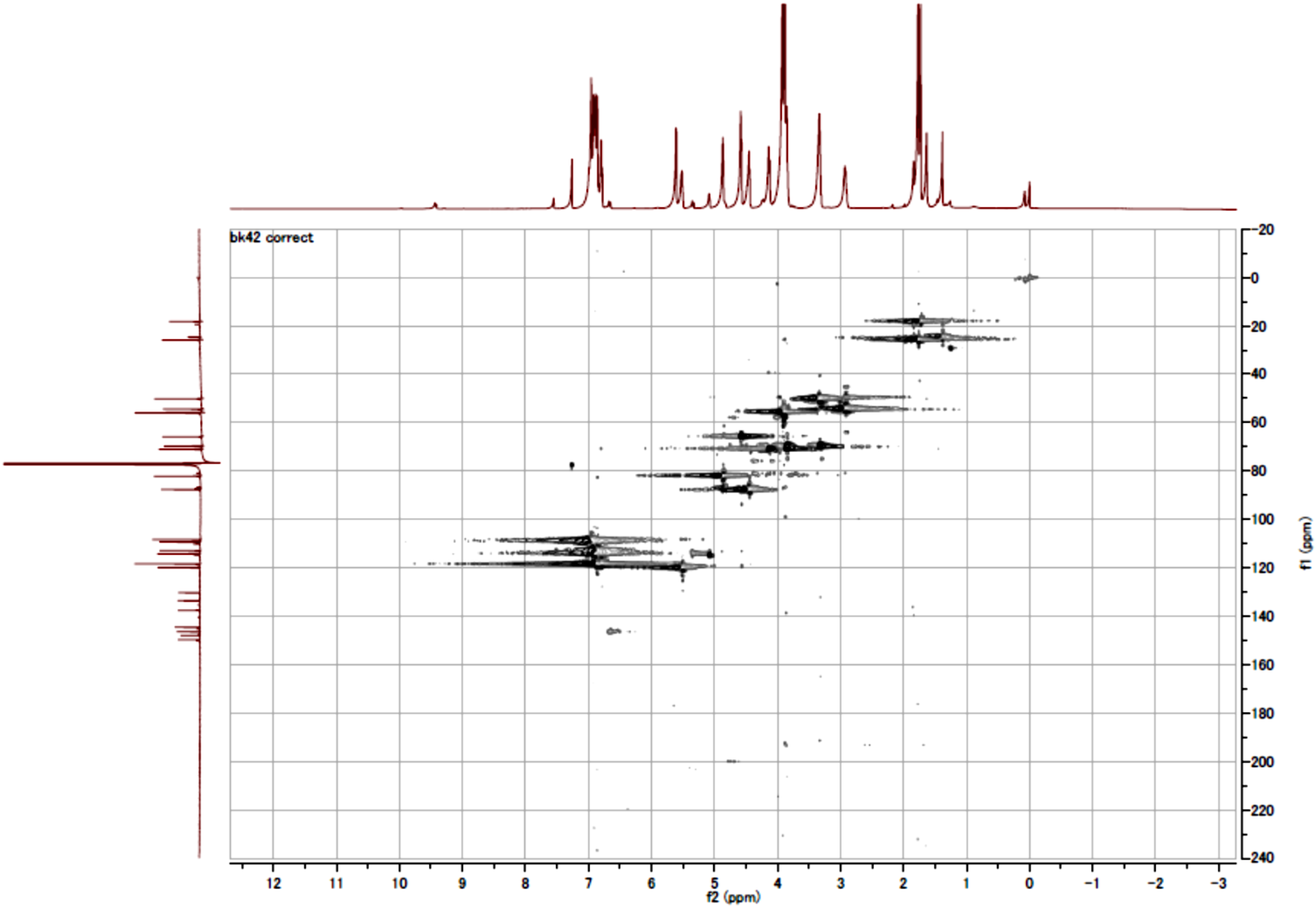

Figure S2: HSQC spectrum of planispine A.

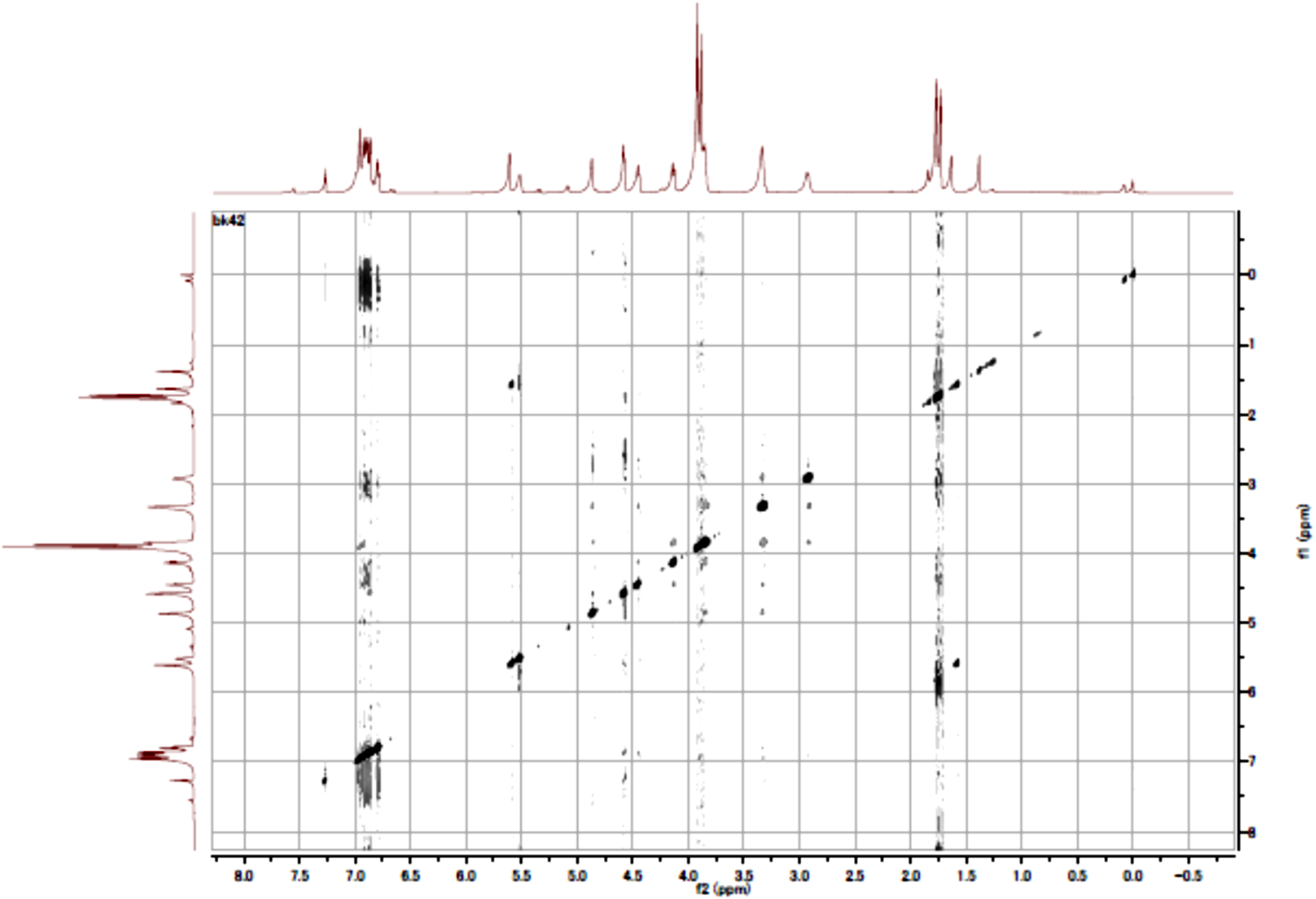

Figure S3: NOESY spectrum of planispine A.
